# NIH funding trends to US medical schools from 2009 to 2018

**DOI:** 10.1371/journal.pone.0233367

**Published:** 2020-06-01

**Authors:** Paige Noble, Patrick Ten Eyck, Robert Roskoski, J. Brooks Jackson

**Affiliations:** 1 Department of Pathology, Carver College of Medicine at the University of Iowa, Iowa City, Iowa, United States of America; 2 Institute for Clinical and Translational Science at the University of Iowa, Iowa City, Iowa, United States of America; 3 Blue Ridge Institute for Medical Research, Horse Shoe, North Carolina, United States of America; University of Texas Southwestern Medical Center at Dallas, UNITED STATES

## Abstract

Total NIH funding dollars have increased from 2009–2018. We questioned whether this growth has occurred proportionately around the country and throughout allopathic medical schools. Therefore, we compared the trend in NIH grant funding from 2009 to 2018 for United States allopathic medical schools among historically top-funded schools, private and public schools, and by region of the country. Changes in both unadjusted and real funding dollars over time revealed a significant difference. Region was the only significant factor for mean percent change in funding from 2009–2018, with the Western region showing a 33.79% increase in purchasing power. The Northeastern region showed a -6.64% decrease in purchasing power while the Central and Southern regions reported changes of 2.46% and -6.08%, respectively. The mean percent increases were more proportional and nonsignificant in the public vs. private institutions comparison, at -3.41% and 4.75%, respectively. Likewise, the top-funded institutions vs. other institutions comparisons demonstrated modest, nonsignificant differences. However, although the relative changes might be proportional, the absolute increases evidence a pattern of growing cumulative advantage that favor the highest-funded institutions and private institutions. The potential consequences of this disproportionate increase include health science education, biomedical research, and patient access disparities in large parts of the country. The NIH and the scientific community should explore potential solutions in its funding models.

## Introduction

Academic medicine is vital to the health of the United States population for training the healthcare providers and leading the advances in medical research. As health care reimbursement rates and state support for public universities have fallen [1], medical schools—especially public medical schools—are under significant financial pressure to adequately support research. Over the last 10 years the number of medical schools have grown and expanded [2], and the National Institutes of Health (NIH) funding for biomedical research has increased [3]. However, it is unclear if the additional funding has been distributed proportionately. Recent publications have raised concerns that NIH support has become increasingly concentrated in the hands of a select group of institutions, exacerbating funding disparities rather than bridging them[4,5].

Questions over the equity of research funding distribution are a longstanding concern. As early as the 1970s, Merton put forward the concept of cumulative advantage to explain why more prominent scientists received disproportionate credit for their contributions to science—propelling their careers to even greater heights—while other scientists' comparable contributions might achieve only limited recognition[6]. In colloquial terms, the principle is commonly summarized as “success breeds success.” The phenomenon has been further characterized through mathematical models and the scope broadened to encompass sociological patterns such as social mobility, and patterns of institutional success [7,8]. In 1979, the National Science Foundation launched the Experimental Program to Stimulate Competitive Research (EPSCoR) in response to concerns that funding patterns consistently overlooked several states, leading to a substantial cumulative disadvantage. The program was intended to bolster research and research infrastructure in states that had been historically underfunded. The program has persisted, and in the modern era, EPSCoR program has been adopted by other agencies including the National Aeronautics and Space Administration. In 1993, a similar program was created by the NIH: the Institutional Development Award Program (IDeA) [9]. IDeA was designed to strengthen university-based research infrastructure in historically underfunded states and territories, and to promote access to research training opportunities for students in those states.

How successful has IDeA been in increasing competitiveness of investigators in those historically underperforming states? Limited studies have been published on specific programs funded through IDeA. These studies offer a mostly positive interpretation of the impact on the program on training opportunities for undergraduate students, and access to clinical trials for residents in qualifying states [10–17]. However, in the program’s more than twenty year history, the number of states eligible for support has not decreased, and many contend that significant funding inequality persists. Therefore, to evaluate a current trajectory of NIH grant funding disparities, we compared the trend in NIH grant funding from 2009 to 2018 for United States allopathic medical schools among historically top-funded schools, private and public schools, and by region of the country.

## Materials and methods

For comparisons, we obtained data from the Blue Ridge Institute for Medical Research database, which lists the NIH grant funding (not including research and development contracts or American Recovery and Reinvestment Act funding) for United States allopathic medical schools accredited by the Liaison Committee on Medical Education [18]. Funding data was obtained from the years 2009, 2012, 2015, and 2018. We examined the proportional change in NIH grant funding received by schools from 2009 to 2018. Comparisons were made between higher and lower funded institutions, region, and public or private status. Our three highly funded groupings are based on the top 10, 20, and 50 funded US medical schools in 2009 To adjust for inflation, we used the Biomedical Research and Development Pricing Index (BRDPI) to convert dollar amounts to constant 2009 dollars to assess relative changes in purchasing power. Regional NIH funding was compared using the four regional groups as defined by the Association of American Medical Colleges (Southern, Northeastern, Central, and Western). Comparisons of proportional change in funding between 2009 and 2018 were performed based on mean change in funding using univariate generalized linear models with a log link function due to the right-skewed nature of the data.

## Results and discussion

Overall and stratified funding totals are provided in Tables 1 and 2. Total NIH funding for all 123 medical schools with funding in 2009 increased from $11.5B to $14.3B, with a mean increase of 24.2% (p < 0.0001). The top 10 schools in 2009 account for 29.7% of all NIH funding and saw a 20.9% mean increase, which was smaller than the bottom 113 who had a mean increase of 24.4% (p = 0.8385). The top 20 schools in 2009 account for 50.6% of all NIH funding and saw a 27.2% mean increase, which was larger than the bottom 103 who had a mean increase of 23.6% (p = 0.7843). The top 50 schools in 2009 account for 83.1% of all NIH funding and saw a 25.4% increase, which was larger than the bottom 73 who had a mean increase of 23.3% (p = 0.8329). The 50 private schools had an 18.2% mean increase compared to 28.2% of the 73 public schools (p = 0.3071). The only significant factor for differences in change over 2009–2018 was region (p = 0.0149). Central, Northeast, Southern, and Western regions had 25.4%, 14.3%, 15.0%, and 63.8% increases in funding, respectively.

**Table 1:** NIH funding over time and tests for significant change within- and between-groups.

Group	N	2009 Funding	2012 Funding	2015 Funding	2018 Funding	Mean change per Institution (2009–2018)	Mean % change (2009–2018)	Group p-value (2018 vs. 2009)	Growth Ratio (GR)	GR Lower	GR Upper	Between-Group p-value
All	123	$11.52B	$11.79B	$11.67B	$14.25B	$22.21M	24.15%	< .0001				
Top 10	10	$3.47B	$3.49B	$3.45B	$4.20B	$72.40M	20.86%	0.1676	0.9712	0.7335	1.2860	0.8385
Not top 10	113	$8.05B	$8.30B	$8.23B	$10.06B	$17.77M	24.44%	< .0001				
Top 20	20	$5.75B	$5.91B	$6.02B	$7.24B	$74.29M	27.20%	0.0132	1.0295	0.8362	1.2674	0.7843
Not top 20	103	$5.77B	$5.88B	$5.65B	$7.01B	$12.10M	23.56%	< .0001				
Top 50	50	$9.51B	$9.72B	$9.73B	$11.95B	$48.65M	25.39%	0.0002	1.0170	0.8699	1.1889	0.8329
Not top 50	73	$2.01B	$2.07B	$1.94B	$2.31B	$4.10M	23.30%	< .0001				
Private	50	$6.04B	$6.21B	$6.29B	$7.73B	$33.83M	18.23%	0.0062	0.9221	0.7892	1.0774	0.3071
Public	73	$5.48B	$5.58B	$5.38B	$6.52B	$14.25M	28.21%	< .0001				
Central	31	$2.58B	$2.62B	$2.57B	$3.13B	$17.81M	25.41%	0.0025				0.0149
Northeast	33	$3.65B	$3.70B	$3.66B	$4.54B	$26.96M	14.27%	0.0662				
Southern	42	$2.89B	$2.89B	$2.81B	$3.38B	$11.47M	14.96%	0.0303				
Western	17	$2.40B	$2.58B	$2.63B	$3.20B	$47.55M	63.75%	< 0.0001				

Growth ratio for dichotomized groups is the top over the bottom (e.g., Private / Public).

*p*-values were calculated using generalized linear models.

**Table 2:** NIH adjusted funding over time and tests for significant change within- and between-groups.

Group	N	2009 Funding	2012 Funding	2015 Funding	2018 Funding	Mean change per Institution (2009–2018)	Mean % change (2009–2018)	Group p-value (2018 vs. 2009)	Growth Ratio (GR)	GR Lower	GR Upper	Between-Group p-value
All	123	$11.52B	$10.99B	$10.24B	$11.64B	$1.00M	1.43%	0.7162				
Top 10	10	$3.47B	$3.25B	$3.02B	$3.43B	-$4.37M	-1.25%	0.9267	0.9712	0.7335	1.2860	0.8385
Not top 10	113	$8.05B	$7.73B	$7.21B	$8.22B	$1.48M	1.67%	0.6849				
Top 20	20	$5.75B	$5.51B	$5.28B	$5.91B	$8.06M	3.92%	0.6917	1.0295	0.8362	1.2674	0.7843
Not top 20	103	$5.77B	$5.48B	$4.96B	$5.73B	-$0.37M	0.95%	0.8251				
Top 50	50	$9.51B	$9.06B	$8.53B	$9.76B	$4.93M	2.45%	0.6938	1.0170	0.8699	1.1889	0.8329
Not top 50	73	$2.01B	$1.93B	$1.71B	$1.89B	-$1.69M	0.74%	0.8848				
Private	50	$6.04B	$5.78B	$5.52B	$6.32B	$5.54M	4.75%	0.3593	0.9221	0.7892	1.0774	0.3071
Public	73	$5.48B	$5.20B	$4.72B	$5.33B	-$2.10M	-3.41%	0.5707				
Central	31	$2.58B	$2.44B	$2.25B	$2.56B	-$0.69M	2.46%	0.7458				0.0149
Northeast	33	$3.65B	$3.45B	$3.21B	$3.71B	$1.78M	-6.64%	0.3442				
Southern	42	$2.89B	$2.69B	$2.47B	$2.76B	-$3.24M	-6.08%	0.3302				
Western	17	$2.40B	$2.40B	$2.31B	$2.62B	$13.05M	33.79%	0.0040				

Growth ratio for dichotomized groups is the top over the bottom (e.g., Private / Public).

*p*-values were calculated using generalized linear models.

Although region was the only significant factor for difference in mean percent change from 2009 to 2018, there are large differences between the mean changes in funding for programs. The mean change in funding for a Top 10 institution was $72,398,413 per institution as compared to a mean change of $17,767,564 for all other institutions [Table 1]. After adjusting for inflation, no significant change in purchasing power is observed [Table 2]. A similar, if not more favorable, trend is seen in the Top 20 and Top 50 stratifications, a pattern of preserved resource accumulation at the highest-funded institutions. For public schools, the mean change was $14,250,072 per institution, whereas the mean change for private schools in the same time period was $33,829,272 per institution [Table 1]. Adjusting for inflation, this represents a -3.41% decrease in purchasing power for public institutions from 2009–2018, and a 4.75% increase in purchasing power for private institutions [Table 2]. The change is not statistically significant. However, in the context of decreasing state support for most public universities [1] and a larger share of Medicaid and Medicare patients with declining reimbursements cared for by them, there emerges a pattern of potential financial strain to the research mission of public institutions.

In the regional analysis, we see that mean changes per institution track with mean funding observed in 2009. The western region dominated the rankings in 2009 with $2.397 billion total for 17 institutions counted, and experienced the greatest mean change per institution at $47,548,706. The Northeast region followed with $3.651 billion total for 33 medical schools in 2009, and experienced the second highest mean change at $26,959,449per institution. The Central and Southern regions lagged behind in 2009 with $2.581 billion for 31 institutions and $2.894 billion for 42 institutions, respectively. The mean changes for the Central and Southern regions were $17,805,975 and $11,470,091 per institution, respectively [Table 1]. Adjusted for inflation, these trends translate to an increase in purchasing power of 33.71% for the Western region, a decrease in purchasing power of -6.64% for the Northeast region, and changes of 2.46% for the Central and -6.08% for the Southern region [Table 2]. Of note, there are currently 24 states and territories eligible for NIH IDeA funding. Broken down by region, the southern region has the most IDeA eligible states with allopathic medical schools (8), followed by the Central region (4). The northeastern and western regions both have 3 IDeA eligible states with allopathic medical schools.

Taken together, the trends in mean funding increases suggest a pattern of cumulative advantage. Proportional growth is similar in most categories, but in absolute increases the rich grow steadily richer. Continued indefinitely, the country is very likely to experience an escalating disparity in research resource allocation, and it is questionable whether lower funded institutions will continue to be able to compete. This disproportionate increase is somewhat predictable, given the large populations and number of medical schools on the coasts and consequent greater number of faculty. Nevertheless, if current trends persist, concerns arise that someday there will only be a few dozen, mostly private, well-funded medical schools performing research, while the rest primarily focus on education. The concentration of NIH funded research at a small percentage of medical institutions in limited geographic areas would limit access to research training to fewer medical and graduate students, while geographic limitations would compromise patient access to promising experimental therapies.

One could reasonably assert that NIH funding should be awarded based on the best scientific proposals regardless of the geography and status of a public or private institution. However, this perspective ignores the impact of cumulative advantage on research infrastructure, and thus the systemic disadvantages that investigators from historically underfunded institutions face when competing for funding. This perspective also does not address the inevitable wasted intellectual capital when talented young students and faculty are unable to access adequate research training and support. In addition, scientific productivity of highly-funded institutions decreases with increased research funding as measured by the number of publications and their aggregate citation ratios per dollar of research project grant funding [4,5].

This scenario is not likely to be in the best interests of the country, training of medical students, and patient care, but what are potential remedies? On the one hand, it seems unwise to penalize well-supported institutions leading scientific advances. On the other hand, bolstering regional NIH support for building and sustaining research infrastructure in those underfunded areas of the country will likely benefit patient access to new therapies, and training opportunities for students, especially in less densely populated regions of the country. Possible initiatives could include mandatory allocations of the National Center for Advancing Translational Sciences Clinical and Translational Science Awards program funding[19], or institutional based K-awards for rural and/or public institutions in IDeA program states [20,21].

In our review of the literature on IDeA initiative outcomes, we were heartened to see consistent reports of positive impacts on undergraduate students and communities from increased access to research opportunities and clinical trials.^10-17^ We found no literature directly evaluating the impact of IDeA initiatives on medical education, but it is reasonable to assume that medical students and trainees would experience a similar, if not greater, benefit from access to research opportunities. Thus, we do not interpret the lack of decline in states and territories qualifying for IDeA support as evidence of a failed program. The research infrastructure of any given institution is the sum of a complex set of factors, including historic state and NIH support, and the economic performance of the state. Cumulative advantage or disadvantage is accrued over decades of relative investment. Ensuring access to high quality research training and support for students and investigators in all states necessitates a similar longitudinal commitment.

## Conclusions

In conclusion, we found that NIH funding increased significantly to both private and public allopathic medical schools, as well as to top 10, 20, and 50 funded medical schools between 2009 and 2018 in absolute terms, but not adjusted for inflation. However, by region of the country, there was a significant difference in mean percent change between regions with the Western region of the country receiving significantly more in absolute and mean percent change in funding even adjusted for inflation. If this trend in concentration of NIH funding persists, basic science and clinical research opportunities for faculty, students and residents could be significantly decreased in many medical schools and regions of the country. Potential consequences of this disproportionate change are in the areas of health science education, biomedical research, and patient access disparities encompassing large parts of the country. The NIH and the scientific community should explore potential solutions in its funding models.
